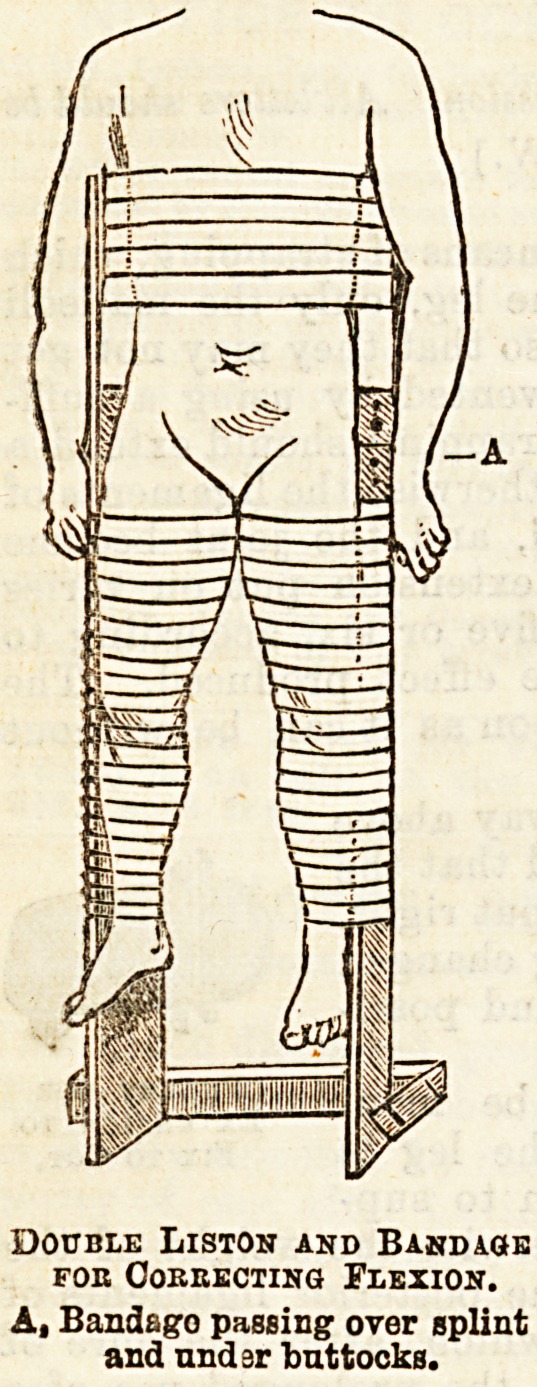# The Treatment of Morbus Coxae

**Published:** 1893-02-11

**Authors:** 


### THE HOSPITAL CLINIC.

[*The Editor will be glad to receive offers of co-operation and contributions from members of the profession. All letters should be addressed to* THE EDITOR, THE LODGE, PORCHESTER SQUARE, LONDON, W.]

### THE ALEXANDRA HOSPITAL FOR DISEASE OF THE HIP.

### THE TREATMENT OF MORBUS COXAE.

In the treatment of morbus coxae at the Alexandra Hospital for Diseases of the Hip one principle is kept constantly in view, and this is, that the most important point to be secured is, as far as possible, complete rest to the joint. We shall first speak of the general treatment applicable to all cases, next of the treatment of acute cases with or without abscess formation, then of the correction of deformity, and, lastly, of the subsequent treatment after leaving the hospital.

All the patients at the Alexandra are nursed on a hard mattress without springs, and under the mattress are placed fracture boards, so that as far as possible a flat firm support is obtained, and although patients may have to lie on this for from one to two years, no difficulty is experienced in preventing the formation of bedsores. Another important point is the smallness and lowness of the pillow, so that the head is not much raised from the horizontal. Then in order to prevent the patient from rising in bed (for they are, it need hardly be said, almost all children) the braces are used, which consist of a webbing strap passing across the chest to two webbing loops through which the arms are passed, then through these again is put a strap which, passing under the patient's back, is fixed under the bed; by this means the child is effectually prevented from rising, while at the same time the chest is not confined at all. Another advantage of this method is that very soon, when a child finds it cannot rise, it ceases to try to do so.

In acute cases there is one line of treatment which is practically always adopted, namely, the application of extension. This, by preventing the pressure of the diseased joint surfaces against one another, at once reduces the pain and irritation, and especially the night startings which are so distressing; but in order to do this, the weight must be properly applied.

The first thing done is to put a long Liston splint on the opposite leg. This, as it has a cross piece below the foot, prevents the child from rolling over and getting the extension pulling in a wrong direction.

EXTENSION APPLIED IN THE AXIS OF THE DISEASED LIMB.

Before the extension is put on, the true position in which the diseased limb is fixed is found by placing the two anterior superior spines on the same level, and then completely overcoming the lordosis by flexing the sound limb fully on the abdomen; the limb then is landed up into its abnormal position by means of pillows or otherwise and the extension put on so as to pull in the direction of its axis in its abnormal position (see diagram). If this be not done, one of the effects of putting on the extension will be to increase and not diminish the pressure of the joint surfaces against one another, and so increase the trouble.

The extension is put on by means of strapping, which is best applied directly to the leg, only the malleoli being covered with bandage, so that they may not get rubbed, and this is also prevented by using a sufficiently wide spreader. The strapping should extend a little way above the knee, as otherwise the ligaments of that joint would get stretched, and the joint become rather loose. The amount of extension put on varies from about two pounds up to five or six, according to the size of the child and the effect produced. The weight is always reduced as soon as it can be without causing a recurrence of pain.

By using extension in the way above described, it is generally found that the malposition can be gradually put right, the direction of the pull being changed from day to day, as it is found possible, without causing lordosis.

PULLEY FOR EXTENSION TO FIX TO COT.

Another point must here be mentioned, namely, that when the leg is landed up care must be taken to support it under the knee, as otherwise the weight of the leg will cause stretching of the posterior ligaments of the joint and over-extension, which, after the cure of the hip trouble, it will require the prolonged use of a splint to put right.

Should pus form the abscess is opened with careful aseptic precautions, as soon as its location can be certainly made out, by the presence of fluctuation. Sometimes the abscess is washed out and injected with an iodoform emulsion (made of olive oil and 10 per cent. of iodoform, with a little potassium iodide), the opening being then stitched up closely, when it will sometimes close without further trouble. More often, however, the abscess is simply drained. Corrosive sublimate lotion ((1-2,000) and sal alembroth gauze and wool are the antiseptics which are generally used.

The subsequent treatment of sinuses thus formed is just to allow them to drain, occasionally exploring with a probe, to see if there may not be a small sequestrum which can be removed.

Should the temperature rise, search is made for any fresh collection or want of free drainage, and a fresh opening or counter-opening made. If this is not found to be causing the trouble, the sinus is syringed out daily with corrosive sublimate lotion. No larger operation than sequestrotomy is ever done at this hospital, and if an excision or an amputation becomes absolutely necessary, which is very rarely the case, the patient has to be removed elsewhere.

Of course, the above treatment requires a large amount of time, and as patients are generally kept in until the sinuses are quite healed, they are often inmates of the hospital for a period extending over from six months to two years.

The method of overcoming malposition by means of extension has already been explained; but one or two other methods are also in use, though they are only applicable in cases which have become chronic, and must be carefully watched to see that they do not set up fresh inflammatory mischief.

One way is to keep the patient lying on his face. This much assists in overcoming the flexion, and the effect may be increased by putting some weight on the buttocks, in the shape of sandbags, &c. Another way of treating the flexion is to use a double long Liston splint. A bandage is then put on which passes over the splint and under the buttocks, and then over the splint again. The legs having been previously bandaged to the splint, by pulling the above-described bandage more or less tight, an effect can be produced on the flexion of the limb. (See figure.)

DOUBLE LISTON AND BANDAGE FOR CORRECTING FLEXION.

A, Bandage passing over splint and under buttocks.

To produce an effect on the abduction or adduction the double Liston is also used, and by using pads placed either at the hip or at the leg, and then bandaging, these deformities are overcome; they are, however, much more difficult to treat than the flexion, though this latter, in some cases, cannot be completely overcome—at any rate, without operative interference, such as division of the femur by osteotomy when the disease has become quiescent.

There now only remains to be described the treatment of the patients after they are allowed to get up. This is carried out by means of a Thomas's hip splint, which is too well known to need any description here. It should be made to fit well, by a curve to correspond to the convexity of the buttock, and should extend from the angle of the scapula to a little above the ankle. If there is much shortening, of course a patten on the sound foot is unnecessary, as all that is required is that the foot of the diseased limb should just quite clear the ground.

In this splint the patient at first gets up and is about the ward; so soon as things are found to be in a satisfactory condition, he is sent home; but the friends are carefully instructed as to bringing the patient up to be seen by the surgeon once a month, or once in three months or six months, as may be thought needful. This carefully carried out, supervision of the after-treatment is one of the most important and useful features at this hospital, and is still continued when all splints and apparatus have been laid aside. This is done when a month or two has elapsed after all pain has disappeared.

It goes without saying, that a hospital such as this is always full, so that applicants for admission must of necessity have to wait their turn. In these cases an extension stirrup is sometimes put on at the hospital, and the friends of the patient told how to use it at home, until their turn comes round to come into the hospital for proper supervision.

There can be no doubt that at this hospital most excellent results are obtained, with a very small percentage of deaths, and these almost always, if not always, due to the disease being far advanced before the patient is seen, or to such complications as Potts' disease of the spine, or occasionally general tuberculosis or tubercular meningitis. Phthisis, we believe, is one of the rarest complications.

## Figures and Tables

**Figure f1:**
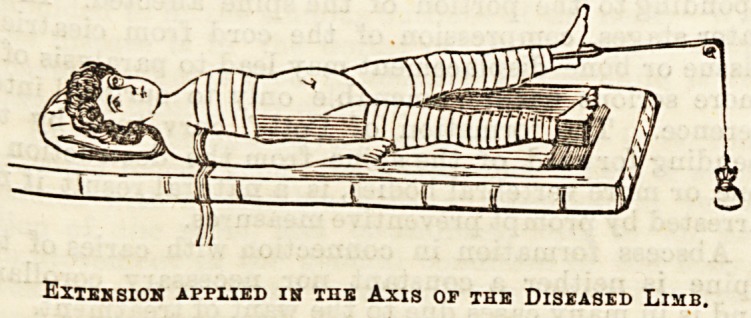


**Figure f2:**
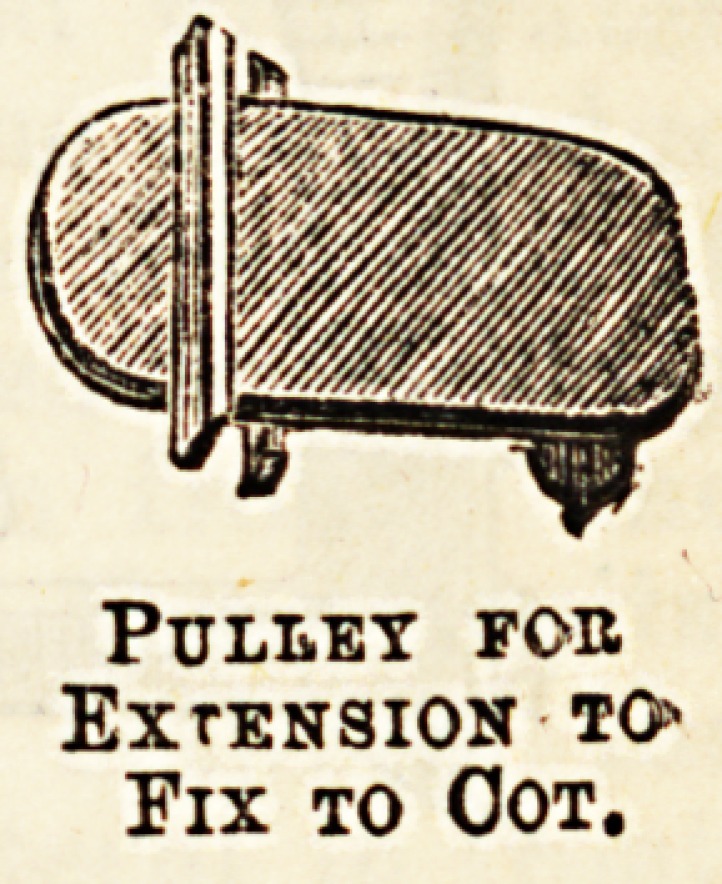


**Figure f3:**